# Prosodic Similarity Effects in Short‐Term Memory in Developmental Dyslexia

**DOI:** 10.1002/dys.1535

**Published:** 2016-10-17

**Authors:** Usha Goswami, Lisa Barnes, Natasha Mead, Alan James Power, Victoria Leong

**Affiliations:** ^1^ University of CambridgeDepartment of PsychologyCambridgeUK

**Keywords:** prosody, phonology, serial recall

## Abstract

Children with developmental dyslexia are characterized by phonological difficulties across languages. Classically, this ‘phonological deficit’ in dyslexia has been investigated with tasks using single‐syllable words. Recently, however, several studies have demonstrated difficulties in prosodic awareness in dyslexia. Potential prosodic effects in short‐term memory have not yet been investigated. Here we create a new instrument based on three‐syllable words that vary in stress patterns, to investigate whether prosodic similarity (the same prosodic pattern of stressed and unstressed syllables) exerts systematic effects on short‐term memory. We study participants with dyslexia and age‐matched and younger reading‐level‐matched typically developing controls. We find that all participants, including dyslexic participants, show prosodic similarity effects in short‐term memory. All participants exhibited better retention of words that differed in prosodic structure, although participants with dyslexia recalled fewer words accurately overall compared to age‐matched controls. Individual differences in prosodic memory were predicted by earlier vocabulary abilities, by earlier sensitivity to syllable stress and by earlier phonological awareness. To our knowledge, this is the first demonstration of prosodic similarity effects in short‐term memory. The implications of a prosodic similarity effect for theories of lexical representation and of dyslexia are discussed. © 2016 The Authors. *Dyslexia* published by John Wiley & Sons Ltd.

## Key Messages

•Children find it more difficult to recall three‐syllable words which have the same prosodic pattern of strong and weak syllables.
•This implies that phonological remediation in dyslexia should include a focus on the stress patterning of strong and weak syllables in words.

## Introduction

Structural characteristics of the phonological lexicon have been shown to affect short‐term recall in both typically developing children and children with developmental dyslexia. Short‐term recall of verbally delivered information is usually measured by serial recall tasks, in which participants are required to report aloud and in the correct order a series of monosyllabic words. Performance in these tasks is assumed to rely on the capacity of a short term ‘phonological loop’ or ‘articulatory loop’ which retains verbal information on a temporary basis (e.g. Baddeley & Hitch, 1974; Gathercole, 2013). Children with dyslexia are usually impaired in tasks requiring them to reflect upon or to manipulate verbal information (Ziegler & Goswami, 2005). For example, they are poor at identifying the constituent sounds in words (phonological awareness tasks), they show impairments in phonological short‐term memory tasks (e.g. digit span), and they are slow when asked to name familiar letters, object names, colours, or digits as quickly as possible (rapid automatized naming tasks, RAN). Yet despite these persistent difficulties, classic effects of lexical structure in serial recall tasks such as the word length effect (Hulme & Tordoff, 1989), the phonological similarity effect (Holligan & Johnston, 1988), and the phonological neighbourhood density effect (Thomson *et al.*, 2005), are all intact in children with dyslexia (unless tasks that exceed memory span are used, Johnston *et al.*, 1987). So despite their phonological difficulties, which psycholinguistically are thought to reflect under‐specified phonological representations for words, children with dyslexia have phonological lexicons that appear to be organized in the same way as the lexicons of typically developing children (see Metsala & Walley, 1998, for a review of lexical organization by typically developing children).

The focus of the current study is the phonological similarity effect: that phonologically similar items are recalled less accurately over short retention periods than phonologically dissimilar items. This is a robust effect in serial recall tasks and is found whether phonological similarity is implemented using rhyming letters (e.g. B, C, D, G, P) versus non‐rhyming letters (e.g. F, H, K, L, N; Salamé & Baddeley, 1986), rhyming words (e.g. *torn*, *horn*, *corn*, *shorn*) versus non‐rhyming words (e.g. *wart*, *buff*, *rest*, *hoot*; Tehan *et al.*, 2001), or words sharing overlapping consonant or vowel phonemes (e.g. *bead*, *peace*, *leaf*, *tease*) versus few or no overlapping phonemes (e.g. *bead*, *pace*, *ledge*, *tab*; Justus *et al.*, 2005). Psycholinguistic models of the phonological similarity effect assume that phonological similarity impedes recall because of the phonemic (or onset‐rime) overlap between serial items (e.g. Nimmo & Roodenrys, 2004; Page *et al.*, 2007). The memory traces for these phonemes or onset‐rime units are thought to interfere with each other in the short‐term store, making the correct order of items difficult to recall. Children with dyslexia are assumed to show robust phonological similarity effects despite their phonological difficulties because they suffer similar trace interference effects. Thus, even though the *capacity* of phonological short‐term memory is impaired in dyslexia, when memory span in children with dyslexia is matched to that of younger children of similar reading level (RL control children), equivalent phonological similarity effects are found (Johnston *et al.*, 1987; Holligan & Johnston, 1988).

The ‘phonological deficit’ in developmental dyslexia holds across languages and orthographies (e.g. Ziegler & Goswami, 2005, for review). Children with developmental dyslexia reliably show weakness in phonological awareness tasks, phonological short‐term memory tasks, and RAN tasks, in all languages so far studied. Phonological awareness tasks measure children's ability to reflect on or manipulate the sound structure of spoken words, and are thought to provide an index of the representational quality of a child's long‐term phonological representations for words in the mental lexicon (e.g. Constable *et al.*, 1997; Swan & Goswami, 1997). The reliable ‘deficit’ in phonological awareness tasks is thought to reflect fine‐grained impairments in word representation. One reason that children with dyslexia show intact effects of aspects of word *structure* in short‐term recall tasks may be that their degraded long‐term memory representations for words are still sufficient to support short‐term retention via ‘redintegration’ effects. Stored phonological knowledge has been shown to aid the ‘redintegration’ or reconstruction of decaying temporary linguistic traces of phonological word forms, thereby facilitating recall (Gupta & MacWhinney, 1997; Hulme *et al.*, 1997; Turner *et al.*, 2004; Schweickert, 1993). Both the size of the activated search set of words in long‐term memory and the phonological distinctiveness of these words from others in the lexicon determine the effectiveness of the redintegration process (Roodenrys *et al.*, 2002). For example, nonwords or unfamiliar words are significantly harder to recall than familiar lexical items, and high‐frequency words are recalled better than low‐frequency words (Roodenrys *et al.*, 2002). Further, nonwords that are more ‘wordlike’ are recalled better than nonwords that are less ‘wordlike’ (Gathercole, 1995). Redintegration effects also seem to be intact in children with dyslexia, despite their phonological awareness problems (Thomson *et al.*, 2005). However, the vast majority of the studies documenting these recall and redintegration effects have used single‐syllable words.

Recently, it has been demonstrated that the phonological ‘deficit’ in developmental dyslexia encompasses *suprasegmental* or prosodic levels of phonology as well as segmental (phonemic) levels. Prosodic patterning is a key structural element of spoken language. Children and adults with dyslexia are reliably impaired in prosodic tasks such as the reiterant speech task (e.g. Kitzen, 2001; Goswami *et al.*, 2010; Goswami and Leong, 2013). In reiterant speech tasks, each syllable in a word is converted into the same syllable (e.g. DEE). This serves to remove most phonetic information while retaining the stress and rhythm patterns of the original words and phrases. Kitzen (2001) converted film and story titles into ‘DeeDees’, so that (for example) ‘Casablanca’ became DEEdeeDEEdee (STRONG weak STRONG weak or SWSW). Sensitivity to syllable stress can also be measured directly, by mis‐stressing words in a same‐different judgement task, for example ‘DIFFiculty – diffICulty’ (this word has primary first syllable stress, SWWW, so the second example is mis‐stressed, with primary stress on the second syllable, WSWW; see Leong *et al.*, 2011). Leong *et al.* (2011) found that English adults with dyslexia were significantly poorer at perceiving lexical stress in this same‐different task compared to controls, and Soroli *et al.* (2010) found a similar result in a same‐different stress judgement task given to French adults with dyslexia (based on pseudowords). Meanwhile, Spanish children with dyslexia were found to be impaired in comparison to age‐matched controls in judging which syllable in a three‐syllable item carried primary stress, for both familiar real words and for pseudowords (Jiminez‐Fernandez *et al.*, 2014).

The prosodic awareness difficulties exhibited by children with dyslexia also appear to persist with development. In a recent longitudinal study of sensitivity to syllable stress patterns in dyslexia, prosodic sensitivity as measured by the DeeDee task at age 9 was significantly related to prosodic sensitivity as measured by the same‐different judgement mis‐stressing task based on four‐syllable words at age 13 (Goswami *et al*., 2013). In this study, the children with dyslexia showed impaired sensitivity to syllable stress compared to *both* reading‐level and age‐matched controls when aged 9 years, and to age‐matched controls only when aged 13 years. When the longitudinal predictors of sensitivity to syllable stress were investigated, and prosodic sensitivity at Time 1 was controlled as the autoregressor, measures of auditory sensitivity to amplitude envelope ‘rise time’ and measures of phonological awareness (rhyme and phoneme awareness) were unique predictors of prosodic sensitivity. Rise times are auditory ‘edges’ or landmarks in the continuous speech signal associated with amplitude (energy) modulations, and potentially help the brain to identify different temporal modulation rates in the speech envelope (Leong & Goswami, 2015). For example, the rise times of successive syllable‐related modulations in the speech envelope are critical linguistic perceptual events that aid parsing (Doelling *et al.*, 2014). Rise times will vary with the phonetic properties of the syllable (e.g. plosive versus glide) and are larger and perceptually more salient when a syllable is stressed (see Goswami & Leong, 2013). Experimentally, difficulties in the accurate perception of rise times are related to less efficient processing of *both* prosodic and sub‐lexical phonology (Goswami, 2015).

Efficient auditory processing of syllable stress and speech rhythm may be particularly important early in the development of children's phonological representations (Goswami & Leong, 2013), and therefore even quite small initial differences in auditory sensitivity to rise time during infancy could affect phonological development as lexical representations are acquired. Rhythmic stress patterns can be perceived while the infant is in the womb, and studies with infants using EEG (electrophysiology) show that sensitivity to native versus non‐native rhythmic stress templates is present by 5 months of age (Weber *et al.*, 2005). The rhythm of stress placement aids infants with segmentation of the speech stream, for example in word finding (e.g. Echols, 1996). Our theoretical focus in the current paper is whether the prosodic impairments that we have identified in children with dyslexia will affect phonological memory in a serial recall task. As children's lexical representations appear to encode prosody less distinctly in dyslexia (Goswami *et al*., 2013), redintegration effects may be less successful when the target items to be recalled have similar prosodic structure. Accordingly, any prosodic similarity effects that may occur in serial recall tasks may be reduced in participants with dyslexia.

To investigate this question, we designed a novel short‐term memory task based on triples of three‐syllable words, a trial length within the short‐term memory capacity of the participants with dyslexia (14‐year‐olds). In the task, we varied whether the stress patterning (strong‐weak syllable alternation pattern) was the same in all three words, as in ‘*masterpiece*, *colourful*, *juvenile*’ (all SWW), or different in each of the three words, as in ‘*indistinct*, *unfriendly*, *occupy*’ (WWS, WSW, SWW). Our hypothesis was that prosodic similarity should exert comparable effects on serial recall as phonemic (or onset‐rime) similarity, with items sharing the same pattern of strong and weak syllables recalled less accurately. Of particular interest was whether our participants with dyslexia would show a phonological similarity effect in this prosodic task. As our participants were drawn from the same longitudinal study reported by Goswami *et al*., 2013, we were also able to investigate whether earlier individual differences in auditory sensitivity to amplitude envelope rise time would predict performance in the prosodic memory task. Other predictors of interest were earlier short‐term memory performance (measured by a serial recall task based on single syllable words), earlier sensitivity to syllable stress, earlier vocabulary development, and earlier phonological awareness skills.

## Methods

### Participants

Fifty‐eight children and teenagers in total participated in the study, 18 teenagers with dyslexia (‘DYS’; 10 males, 8 females), 24 chronological age‐matched controls (‘CA’; 14 males, 10 females), and 16 reading level‐matched controls (‘RL’; 8 males, 8 females). All were participants in a 6‐year longitudinal study of developmental dyslexia that had begun when the children were aged 7 – 8 years (Goswami *et al*., 2011, 2013). The data reported here were collected in Year 6 of the study, when the participants with dyslexia were aged on average 14 years (mean age DYS = 14.8 years, SD 1.1 years; mean age CA = 14.4 years, SD 1.0 years; mean age RL = 11.9 years, SD 0.8 years). Participants comprised all those remaining from the original cohort (see Goswami *et al*., 2013) at the time that the current study was run. The children with dyslexia were originally recruited via learning support teachers, and only children with no additional documented learning difficulties (e.g. dyspraxia, ADHD, autistic spectrum disorder, specific language impairment [SLI]), a nonverbal IQ above 85, and English as the first language spoken at home were included. The absence of additional learning difficulties was based on school reports, discussion with parents, and our own testing. All children (dyslexics and controls) received a short hearing screen using an audiometer. Sounds were presented in both the left or right ear at a range of frequencies (250, 500, 1000, 2000, 4000, 8000 Hz), and all participants were sensitive to sounds within the 20 dB HL range. Dyslexic children were included in the study if they had a statement of developmental dyslexia from their local education authority, and/or showed severe literacy and phonological deficits according to our own test battery. Children were assessed experimentally using the British Ability Scales (BAS, Elliott *et al.*, 1996) and Test of Word Reading Efficiency (TOWRE, Torgesen *et al.*, 1999) standardized tests of reading and nonword reading, and the BAS spelling subtest. They were included in the study if they scored at least 1 standard deviation below the test norm of 100 on at least one of the two reading measures used when the study began (BAS and TOWRE).

Reading was re‐assessed at the current test point using the BAS Reading subtest and the TOWRE real word and nonword subtests. As shown in Table 1, the teenagers with dyslexia continued to show significant deficits in single word reading. They were still matched to the younger RL children for average reading age on the BAS (DYS = 10.7 years, SD 2.2 years; RL = 11.8 years, SD 2.0 years), although a lag is becoming evident in comparison to earlier test points, when the groups were more closely matched in absolute scores (Goswami *et al*., 2013). Receptive vocabulary as assessed by the British Picture Vocabulary Scales (BPVS; Dunn *et al.*, 1997) was also measured, and did not differ between groups, as shown in Table 1. All children had also completed four subscales of the Wechsler Intelligence Scale for Children at the beginning of the study (WISC‐III; Wechsler, 1992): Block Design, Picture Arrangement, Similarities, and Vocabulary. These four scales yielded an estimate of full‐scale IQ (using a formula from those offered by Sattler, 1982, which enables full‐scale IQ [FSIQ] scores to be pro‐rated from different combinations of sub‐scales). There were no significant IQ differences between the groups, also shown in Table 1.

**Table 1:** Participant characteristics by group

	DYS *N* = 18	CA *N* = 24	RL *N* = 16	*One‐way ANOVA F*(*2*,*57*)
Age in years^a^	14.8 (1.1)	14.4 (1.0)	11.9 (0.8)	43.6***
Reading age in years^b^	10.7 (2.2)	14.4 (1.9)	11.8 (2.0)	18.2***
BAS SS^c^	76.9 (12.5)	106.5 (21.4)	101.6 (15.3)	15.9***
TOWRE real words SS^c^	87.1 (8.8)	100.9 (10.1)	101.4 (12.3)	11.4***
TOWRE nonwords SS^c^	79.3 (13.2)	102.1 (10.1)	98.5 (18.3)	15.4***
BPVS SS	102.9 (21.6)	110.2 (16.3)	109.7 (13.3)	1.1
WISC FSIQ^d^	108.1 (10.5)	112.8 (12.4)	105.2 (9.7)	2.4
Phoneme reversal b(out of 20)	7.5 (5.9)	14.5 (4.1)	9.9 (6.2)	9.6***
RAN time a(seconds)	35.0 (7.7)	32.4 (3.6)	41.1 (4.0)	13.0***
1‐Rise threshold e(ms)	78.3 (56.3)	48.2 (22.3)	68.6 (28.9)	*F*(2,55) = 3.5*

Note DYS, participants with dyslexia; CA, chronological age matched controls; RL, reading level matched controls; BAS, British Ability Scales; SS, standard score; TOWRE, Test of Word Reading Efficiency; BPVS, British Picture Vocabulary Scales (receptive vocabulary); WISC FSIQ, Wechsler Intelligence Scale for Children Full‐Scale Intelligence Quotient; RAN, Rapid Automatized Naming. Standard deviations are shown in parentheses.

* *p* < .05.

** *p* < .01.

*** *p* < .001.

a CA = DYS < RL.

b CA > DYS = RL.

c RL = CA > DYS.

d Measured in first year of longitudinal study and pro‐rated.

e DYS = RL > CA.

### Tasks

- *Prosodic Short‐Term Memory Task*

A novel task was designed for this study in order to examine possible prosodic effects in short‐term memory. The task was an adaptation of the classic phonological short‐term memory task based on three or four items for immediate recall (e.g. Thomson *et al.*, 2005). Here, three items were selected for each trial from the Celex database, each comprising three syllables, selecting words which were deemed likely to be familiar to teenagers. The triplets of three‐syllable words either had the same (Same Stress) or a different (Different Stress) prosodic stress pattern, and triplets were matched as closely as possible for spoken frequency. Care was taken to ensure that syllables did not repeat within a triple, for example frequent prefixes like ‘dis’ and suffixes like ‘tion’ would only appear once in any given triple. Our calculations using Celex showed that, of the three‐syllable English words in the database, approximately 57% had primary stress on the first syllable, 38% had primary stress on the second syllable, and 5% had primary stress on the third syllable. However, it was difficult to generate triples of words with primary stress on the second syllable without repeating syllables within the words. We therefore decided to utilize the most and least frequent rhythmic stress templates within the Same Stress condition.

For triplets with the Same Stress pattern, identical stress patterning was thus achieved either by all items having primary stress on the first syllable (e.g. ‘*melody*’, ‘*treasurer*’, ‘*clinical*’) or all items having primary stress on the third syllable (e.g. ‘*disbelief*’, ‘*reassure*’, ‘*comprehend*’). Words with primary stress on the third syllable also had secondary stress on the first syllable. For triplets with a Different Stress pattern, each of the three words had primary stress on a different syllable (e.g. ‘*bearable*’ (*1st*), ‘*assertive*’ (*2nd*), ‘*undermine*’ (*3rd*)). For the Different Stress condition, the three stress patterns were arranged in all possible orders (1,2,3; 2,3,1; 3,2,1). Items in the Same Stress and Different Stress conditions were also matched for average word frequency (Same Stress, mean triple frequency 13.6; mean item frequency 4.5; Different Stress, mean triple frequency 13.9; mean item frequency 4.7). There were 18 Same Stress trials and 18 Different Stress trials. Item triples and item frequencies were also matched within the two types of Same Stress trial (SWW: mean triple frequency, 13.9; mean item frequency 4.6; WWS: mean triple frequency 13.3; mean item frequency 4.4). The full list of stimuli is included as Appendix A.

The test was administered in the same way as the classic short‐term memory task that uses single syllable words, using digitized speech created from a native female speaker of standard Southern British English. The participants listened to each triplet of words presented by computer and were asked to repeat them back to the experimenter in an identical order. They also completed two practice trials before performing the experimental task, during which feedback was given. Four fixed orders of trial presentation were used, counterbalanced across participants, with trial type (Same Stress, Different Stress) randomly mixed. The number of trials recalled correctly (requiring the order of items to be preserved) and the number of words recalled correctly (accurate item recall in any order) were the two dependent variables used in further analysis.

*Other linguistic measures*

Two other tasks, a phoneme reversal task and a measure of rapid automatized naming (RAN), were also administered at the current test point, as shown in Table 1. The phoneme reversal task was created for this study using digitized speech created from the same native female speaker of standard Southern British English, and was an adaptation for British English of a task reported by Johnson *et al.* (2011) in American English. Participants heard a monosyllabic word, for example ‘*cat*’, and were asked to reverse the order of the sounds to make a new real word, ‘*tack*’. Participants were told ‘You are going to hear some words. Each time you hear a word I want you to think about the *sounds* in the word. So, for example, the sounds in “cat” are /k/, /a/, /t/. I would like you to turn the sounds around to make a new word. So, if we turn /k/, /a/, /t/ around we get /t/, /a/, /k/ ‐ “tack”. You need to say this new word back to me. First, we will try some examples.’ The participants were given three practice trials with feedback (*cat – tack*, *aim – may*, *sell – less*). The subsequent experimental task comprised 20 trials without feedback. Scores out of 20 were used in the analyses.

In the RAN task, participants were asked to name line drawings of two sets of familiar objects (first set: cat, shell, knob, zip, thumb; second set: web, fish, book, dog, cup; see Richardson *et al.*, 2004). For each set, they were first introduced to the names of the pictures and then shown a page with the same pictures repeated 40 times in random order. They were then asked to produce the names as quickly as possible. Average naming speed across the two lists in seconds was used in the analyses.

Various tasks from the previous year's test battery were also used in the correlation and regression analyses. The first was a *phoneme deletion* task. In the phoneme deletion task, the children listened to nonword stimuli and were asked to delete a target sound, e.g. ‘Please say “starp” without the “p”’. The sounds to be deleted were either initial, medial, or final phonemes, and in each case the deletion resulted in a real word. The task comprised 20 trials. The second was a classic *phonological short‐term memory task*, in which children heard four monosyllabic consonant–vowel–consonant nonwords presented by computer through headphones (e.g. *rell*, *kide*, *tave*, *nug*; adapted from a task used in Thomson *et al.*, 2005 in which both real words and nonwords were utilized). The children were required to repeat back the words as spoken. Twenty trials were presented in random order, 10 comprising items with rimes drawn from dense phonological neighbourhoods, and 10 trials comprising items with rimes drawn from sparse phonological neighbourhoods. The total number of items reported correctly out of 80 was used in the current analyses. A *syllable stress judgement task* from the previous year's test battery was also used (see Goswami *et al*., 2013). In this task, participants listened to a four‐syllable word pronounced twice, and made a same‐different judgement about stress pattern. For example, for the word pair ‘*DIfficulty–diFFIculty*’ a ‘different’ judgement was required. The task was based on 10 four‐syllable words with rhythmic stress templates that had first syllable stress (SWWW, such as *caterpillar* and *difficulty*) and 10 four‐syllable words with rhythmic stress templates that had second syllable stress (WSWW, such as *maternity* and *ridiculous*). The words were selected on the basis of syllable structure (no consonant clusters in the first two syllables), spoken and written frequency and overall familiarity, and did not have alternative pronunciations. All items were produced naturally by a native female speaker of British English and recorded for computerized presentation using Audacity and Praat software (Boersma & Weenink, 2010). Children's average performance (d') across the two stress templates (SWWW, WSWW) was the dependent variable in the current analyses. The British Picture Vocabulary Scales had also been administered a year previously, as had the Rise Time task described next.

*Auditory rise time processing*

A psychoacoustic task assessing auditory thresholds for amplitude envelope rise time, administered twice, was used to assess basic sensory processing, see Table 1 (see also Goswami *et al*., 2011, 2013). The task was re‐programmed in Presentation for the current study by Alan Power. The stimuli were presented binaurally through headphones at 75 dB SPL. Earphone sensitivity was calculated using a Zwislocki coupler in one ear of a KEMAR manikin (Burkhard & Sachs, 1975). The rise time task used a cartoon ‘Dinosaur’ threshold estimation interface originally created by Dorothy Bishop (Oxford University). An adaptive staircase procedure (Levitt, 1971) using a combined 2‐down 1‐up and 3‐down 1‐up procedure was used, with a test run terminating after eight response reversals or the maximum possible 40 trials. The threshold was calculated using the measures from the last four reversals. This indicated the smallest difference between stimuli at which the participant could still discriminate with a 79.4% accuracy rate. The participants were assessed individually in a quiet room within their school or at home. A rigorous practice procedure (five trials) was applied prior to the presentation of the experimental stimuli. Rise time sensitivity was measured in an AXB paradigm using three 800 ms tones presented with 500 ms ISI, where one of the comparison stimuli (A or B) had the same rise time as the standard (X), while the other did not. The second tone was always the standard tone, with a 15 ms linear rise time envelope, 735 ms steady state, and a 50 ms linear fall time. Either the first or third tone was identical to this standard, whereas the third or first tone varied the linear rise time envelope along a continuum, with the longest rise time being 300 ms. The child had to select the sound that was different. This task was administered twice, in order to increase threshold reliability (see Boets *et al.*, 2011). Mean performance across the two assessments was used in the analyses.

## Results

Performance in the prosodic short‐term memory task by group is shown in Table 2, with performance shown separately for Same Stress trials and Different Stress trials. To investigate potential prosodic similarity effects without the serial ordering requirement, the number of individual words recalled correctly in the Same Stress trials and the Different Stress trials was also computed, and is also shown in Table 2. If phonological similarity operates at the prosodic level, then items with different prosodic patterns (Different Stress items) should be remembered better than items with similar prosodic patterns (Same Stress items), for both the trials correct measure and the items correct measure. Inspection of Table 2 suggests that this was indeed the case, for all participant groups (DYS, CA, RL).

**Table 2:** Performance on the prosodic short‐term memory task by group

	DYS *N* = 18	CA *N* = 24	RL *N* = 16
Same Stress Trials (out of 18)	9.4 (0.95)	14.5 (0.83)	10.4 (1.0)
Different Stress Trials (out of 18)	11.0 (4.8)	15.5 (2.4)	11.4 (5.2)
Same Stress # words (out of 54)	41.2 (8.7)	49.4 (3.9)	42.9 (7.6)
Different Stress # words (out of 54)	43.7 (8.3)	51.0 (3.1)	44.8 (8.4)
Same Stress Trials, first syllable stress (out of 9)	4.39 (0.50)	7.13 (0.43)	4.50 (0.53)
Same Stress Trials, third syllable stress (out of 9)	5.10 (0.55)	7.42 (0.47)	5.93 (0.58)
Same Stress Trials, first stress # words (% correct)	73.3% (16.0%)	91.2% (8.0%)	76.6% (14.6%)
Same Stress Trials, third stress # words (% correct)	79.2% (18.1%)	91.7% (8.8%)	82.2% (17.0%)
Different Stress # words (% correct)	81.0% (15.3%)	94.4% (5.7%)	82.9% (15.6%)

Note Standard deviations in parentheses.

Inspection of the distributions showed a mild positive skew (<2) for some variables, accordingly we also ran all the ANOVAs that we report below on log‐transformed data. All statistical effects were the same, with similar effect sizes; hence, here we report the analyses for the raw data only. To explore whether the prosodic similarity effect was significant when serial order was preserved, a 3 × 2 (Group [DYS, CA, RL] × Condition [Same Stress, Different Stress]) repeated measures ANOVA was run in SPSS (Statistical Package for the Social Sciences, version 13, IBM Corp), taking the number of trials recalled correctly as the dependent variable. The ANOVA showed a significant main effect of condition, *F*(1,55) = 12.9, *p* < .001, *ηρ^2^* = .190, and a significant main effect of group, *F*(2,55) = 9.3, *p* < .001, *ηρ^2^* = .252, but no significant interaction, *F*(2,55) = 0.44, *p* = .65, *ηρ^2^* = 0.016. The main effect of condition reflected the fact that all groups showed better memory for the items with different stress patterns than for the items with the same stress pattern. Hence, contrary to our initial hypothesis, the participants with dyslexia did not show a reduced prosodic similarity effect. Post‐hoc tests (Tukeys) showed that the main effect of group arose because the CA participants remembered significantly more trials correctly than either the RL or DYS participants, who did not differ (CA vs RL, *p* = .006, mean difference [MD] = 4.1, standard error [SE] = 1.3; CA vs DYS, *p* = .001, MD = 4.8, SE = 1.2; RL vs DYS, *p* = .87, MD = 0.7, SE = 1.3). To check whether a phonological similarity effect would also be found for the number of trials recalled correctly when the requirement to remember item order was removed, a second 3 × 2 (Group [DYS, CA, RL] × Condition [Same Stress, Different Stress]) ANOVA was run, taking the number of words recalled correctly as the dependent variable. The ANOVA showed a significant main effect of condition, *F*(1,55) = 17.2, *p* < .001, *ηρ^2^* = .238 (different items recalled better than similar items), and a significant main effect of group, (2,55) = 8.7, *p* < .001, *ηρ^2^* = .239, but again, no significant interaction, *F*(2,55) = 0.38, *p* = .69, *ηρ^2^* = 0.013. Post‐hoc tests showed that the CA controls again had significantly better memories than the other two groups (CA vs DYS, *p* = .001, MD = 7.7, SE = 2.0; CA vs RL, *p* = .01, MD = 6.4, SE = 2.1), who did not differ (RL vs DYS, *p* = .81, MD = 1.4, SE = 2.2). Hence, the prosodic similarity effect was also found for the less stringent memory measure, the total number of words recalled correctly. Again, there was no sign of a reduced prosodic similarity effect for the participants with dyslexia.

As will be recalled, the Same Stress items comprised two different types of prosodic similarity, words with primary stress on the first syllable, which are frequent in spoken English, and words with primary stress on the third syllable, which are infrequent (see Table 2). In order to explore whether there were any group differences in performance for the two types of Same Stress item, two repeated measures 3 × 2 ANOVAs were run, taking Trial Type (Same Stress, First Syllable; Same Stress, Third Syllable) as the within‐subjects factor and Group (DYS, CA, RL) as the between‐subjects factor. The number of trials recalled correctly and the percentage of words recalled correctly were the dependent variables.

**Figure 1:**
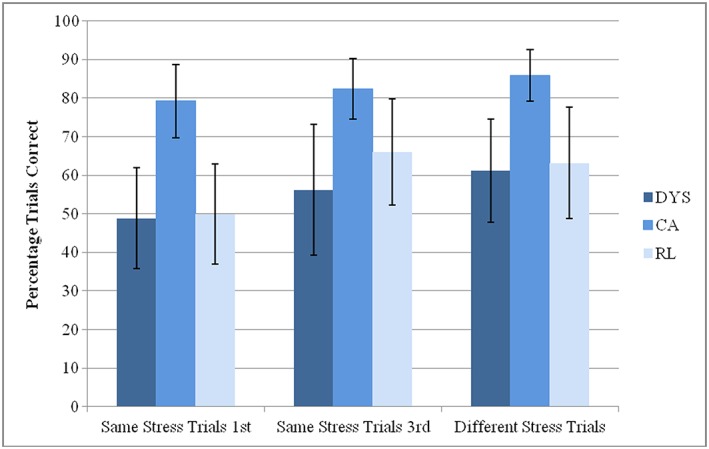
Percentage of trials remembered correctly in the prosodic STM task by trial type and group, when serial order was preserved. Error bars in the chart indicate standard deviations.

The first ANOVA (DV = number of trials) showed a main effect of Group, *F*(2,55) = 9.5, *p* < .001, *ηρ^2^* 0.257 and a main effect of Trial Type, *F*(1,55) = 11.1, *p* < .001, *ηρ^2^* = 0.167, but no significant interaction, *F*(2,55) = 1.9, *p* = .15, *ηρ^2^* = 0.066. The main effect of Trial Type arose because all the participants remembered fewer words correctly in Same Stress trials when the words all had first syllable primary stress. Performance for these first syllable Same Stress trials (e.g. ‘*melody*’, ‘*treasurer*’, ‘*clinical*’) was significantly poorer than performance for Same Stress trials with primary third syllable stress (e.g. ‘*disbelief*’, ‘*reassure*’, ‘*comprehend*’). Tukey's post‐hoc tests showed that the Group effect again arose because both the RL and DYS groups performed significantly more poorly than the CA group (CA vs DYS, *p* = .0001, MD = 2.5, SE = 0.6; CA vs RL, *p* = .007, MD = 2.1, SE = 0.7). Meanwhile, the RL and DYS groups did not differ in performance from each other (RL vs DYS, *p* = .76, MD = 0.5, SE = 0.7). The second ANOVA (DV = % of words recalled correctly) yielded similar findings: a significant main effect of Trial Type, *F*(1,55) = 7.1, *p* = .01, *ηρ^2^* = .114; and a significant main effect of Group, *F*(2,55) = 8.8, *p* < .001, *ηρ^2^* = .241, with no significant interaction, *F*(2,55) = 1.6, *p* = .22, *ηρ^2^* = 0.054. The main effect of Trial Type again arose because the participants remembered more items correctly in Same Stress trials that utilized words with third syllable primary stress. The Group effect again arose because both the RL and DYS groups performed significantly more poorly than the CA group (CA vs DYS, *p* = .001, MD = 4.1, SE = 1.1; CA vs RL, *p* = .012, MD = 3.3, SE = 1.1), while the RL and DYS groups did not differ in from each other (RL vs DYS, *p* = .74, MD = 0.9, SE = 1.2). As previously, therefore, all three groups showed similar patterns of performance. These patterns of performance are shown in Figure 1, which shows performance by item type plotted in terms of the percentage of trials recalled correctly by each group. The data show that rhythmic stress templates that are experienced less frequently appear to interfere with each other less during short‐term retention.

Finally, we investigated potential sources of individual differences in the prosodic memory task. A priori, successful recall should be related to individual differences in sensitivity to syllable stress, to performance in the classic phonological short‐term memory task (no prosodic load), to participants' language and phonological processing skills (measured here by the BPVS, phoneme reversal and RAN), and to rise time discrimination. To assess both concurrent and longitudinal relationships between prosodic memory, vocabulary, phonological awareness, RAN and auditory rise time sensitivity, partial correlation analyses were used (Pearson's), controlling for age and IQ. The results are shown in Table 3.

**Table 3:** Concurrent and time‐lagged correlations between performance in the prosodic STM task and performance in theoretically related tasks

	Prosodic STM # trials correct	Prosodic STM # words correct
Concurrent Rise Time threshold	−.32*	−.36**
Concurrent PA (phoneme reversal)	.46***	−.46***
Concurrent RAN	−.25	−.29*
Concurrent vocabulary (BPVS SS)	.33*	.33*
Phonological STM (12 m earlier)	.71***	.68***
Syllable stress d' (12 m earlier)	.55***	.53***
Rise Time Threshold (12 m earlier)	−.15	−.13
PA (phoneme deletion) (12 m earlier)	.52***	.53***
RAN (12 m earlier)	−.21	−.28*
BPVS SS (12 m earlier)	.42**	0.38*

Note STM, short‐term memory; PA, phonological awareness; RAN, rapid automatized naming; 12 m, 12 months; BPVS SS, British Picture Vocabulary Scales Standard Score.

*** *p* < .001.

** *p* < .01.

* *p* < .05.

As can be seen, performance in the prosodic memory task was significantly related to all of the concurrent predictors with the exception of RAN, which only showed a significant relation for the less stringent measure (number of words correct). The time‐lagged correlations also revealed significant relationships, but for the psycholinguistic measures only. Earlier phonological short‐term memory, earlier sensitivity to syllable stress, earlier phonological awareness (phoneme deletion), and earlier receptive vocabulary all showed significant predictive relationships with prosodic memory. However, earlier rise time sensitivity did not show a significant time‐lagged relationship, and RAN only showed a significant relationship for the words correct measure. Hence, the most consistent contributors to prosodic memory performance were the other phonological and language measures. The auditory processing measure only played a role in *concurrent* prosodic memory abilities, presumably as it was relevant to the accuracy of online stress perception.

Finally, in order to explore the predictors of prosodic memory performance independently of the memory load component of our task, we used performance in the classic phonological short‐term memory task (measured a year earlier) as the autoregressor in multiple regression analyses. If rise time and sensitivity to syllable stress are significant predictors of performance even after controlling for earlier memory abilities, this would suggest that the mental lexicon stores prosodic patterns for individual items, and that the accuracy of the representation of prosody has an additive effect on memory performance, for example via redintegration. A series of four‐step fixed order multiple regression equations were run (*N* = 58). The number of words recalled correctly was the dependent variable in each case. Each equation entered age at step 1 and IQ at step 2, to account for age and IQ differences between participants, then entered performance in the phonological short‐term memory task a year previously at step 3. Concurrent rise time sensitivity, phonological awareness and receptive vocabulary, and rise time sensitivity, vocabulary, phonological awareness, and sensitivity to syllable stress measured a year previously were, respectively, each entered at step 4 in seven separate equations. The results are shown in Table 4.

**Table 4:** *Stepwise regressions showing the unique variance in the Prosodic STM task* (# *words recalled correctly*) *accounted for by sensitivity to syllable stress* (*longitudinal*), *rise time sensitivity*, *phonological awareness*, *and receptive vocabulary* (*all concurrent and longitudinal*), *after controlling for age*, *IQ*, *and earlier short‐term memory performance* (*standardized Beta and R^2^change*)

	Prosodic	STM
Step	Beta	*R^2^*change
1. Age	.280	.079*
2. WISC IQ	.301	.090*
3. STM (classic task, 12 m earlier)	.637	.383***
4. Syllable stress d'(12 m earlier)	−.322	.087**
4. Rise time (concurrent)	−.284	.068**
4. Rise time (12 m earlier)	−.103	.010
4. PA (concurrent)	.271	.064**
4. PA (12 m earlier)	.257	.052*
4. Vocabulary (concurrent)	.262	.059**
4. Vocabulary (12 m earlier)	.259	.056*

Note STM, short‐term memory; WISC IQ, Wechsler Intelligence Scales for Children Full‐Scale Intelligence Quotient; 12 m, 12 months; PA, phonological awareness.

*** *p* < .001.

** *p* < .01.

* *p* < .05.

Table 4 shows that only phonological awareness and receptive vocabulary were both concurrent *and* longitudinal predictors of performance in the prosodic memory task. These measures exerted partly independent effects, as shown in a separate five‐step multiple regression analysis (not shown in Table 4) entering age at step 1, IQ at step 2, earlier short‐term memory performance at step 3, phonological awareness at step 4, and vocabulary at step 5. Here, vocabulary accounted for a unique 7% of additional variance (*p* < .01), on top of the 5% accounted for by earlier phonological awareness. Amplitude envelope rise time perception was only a significant *concurrent* predictor of performance. Sensitivity to syllable stress (not measured concurrently) was another significant longitudinal predictor of performance, accounting for the largest absolute amount of unique variance in prosodic memory (9%). Indeed, taken together, age, IQ, earlier phonological short‐term memory, and earlier sensitivity to syllable stress accounted for almost 64% of the variance in the prosodic memory task. The novel prosodic short‐term memory task developed here appears to be a sensitive measure of individual differences in linguistic performance.

## Discussion

Here, we show that the prosodic structure of words exerts significant effects on short‐term memory performance, with better retention of words that differ in prosodic structure. Significant effects of prosodic similarity were found for teenagers with or without dyslexia and also for typically developing younger children, whether serial word order was preserved at recall or not. All participants found the short‐term recall of phonological information easier when the rhythmic stress templates of three different words were all different, such as strong‐weak‐weak (SWW), WSW, WWS. These prosodic similarity effects were found even though the items used in the prosodic memory task did not share any syllables, ensuring that phonological similarity depended on rhythmic stress patterns.

The data suggest that the prosodic structure of words (the strong–weak syllable patterning of an item) is an important structural variable with respect to the mental lexicon, which appears to store this information on a word‐by‐word basis. The similarity or dissimilarity of strong–weak syllable patterning between items then affects short‐term recall, presumably via supporting redintegration at the supra‐segmental level. This interpretation of our data is suggested by the finding that individual differences in phonological awareness and receptive vocabulary were the only measures that were both concurrent and longitudinal predictors of performance in the analyses that used an autoregressor to control for memory ability (the multiple regressions). Accordingly, both the quality of children's lexical representations (as indexed by their phonological awareness, which would relate theoretically to the phonological distinctiveness with which individual words are stored in the mental lexicon) and vocabulary size (as indexed by the BPVS, which would relate theoretically to the set size being searched) affected the accuracy of short‐term recall. To our knowledge, our task is the first to provide a direct measure of prosodic similarity effects in short‐term memory.

Although participants with dyslexia showed intact prosodic similarity effects, they recalled significantly fewer items correctly than their age‐matched controls, showing impaired short‐term memory capacity despite having had the same number of years of exposure to oral language and being matched at the group level for vocabulary development. If information about rhythmic stress patterning stored in the lexical system helps to reconstruct decaying memory traces for multisyllabic words, then the reduced accuracy of lexical representation of prosody in dyslexia would impair this redintegration process and affect memory capacity. Indeed, we have independent evidence of impaired lexical representation of prosody in related research conducted with the same dyslexic participants tested here. Using electrophysiological (EEG) measures, we have found that the neural representation of speech envelope information (amplitude modulations in the delta band) is atypical in children with dyslexia (Power *et al*., 2013, 2016). Power *et al*., (2013) demonstrated that dyslexic participants showed an earlier preferred phase in the EEG delta band (0 – 3 Hz) compared to CA controls when listening to rhythmic speech, suggestive of neuronal alignment to less informative portions of the signal. Modulations in speech energy in the delta band are thought to support the extraction of linguistic prosody (see Ghitza & Greenberg, 2009). Further, Power *et al*., (2016) demonstrated that when the fidelity of speech envelope encoding was reconstructed from the responses of the neuronal populations using EEG (so that individual stimulus envelopes for spoken sentences were reconstructed from their resultant EEG patterns), then delta band envelope information was encoded significantly less accurately by participants with dyslexia compared to *both* RL and CA controls. The dyslexic participants both encoded this speech information significantly more poorly than the CA and RL controls *and* reported fewer words correctly than the CA controls when reporting the target sentences aloud. Significantly worse neural encoding of the low‐frequency amplitude information in speech at the single sentence level in dyslexia supports the theoretical view that phonological representations for individual words are degraded in the mental lexicon in dyslexia (at least, in the participants studied here).

Comparison of performance for the two types of Same Stress triples utilized for the experiment (SWW, WWS) was also informative. Memory for the most frequent rhythmic stress template (SWW words, the pattern for 57% of three‐syllable words in the lexicon) was significantly poorer than memory for the least frequent rhythmic stress template (WWS words, 5% of three‐syllable words). This effect was found for all the groups, even though Figure 1 is suggestive of attenuation with age. The difference in recall accuracy is again suggestive of redintegration effects, as the activated search set of WWS words would be smaller than the set of SWW words. For prosodic recall, redintegration is hence more successful for the smaller word sets, which would be more distinctive. The significant difference in recall for different types of same‐stress items is suggestive of lexicon‐wide structural effects of rhythmic stress templates. These statistically based structural effects could occur in participants with dyslexia even if the phonological representations for the items themselves were degraded, as the similarity‐based effects would simply operate on differently specified representations.

Finally, when we investigated the predictors of successful memory for three‐syllable words using longitudinal data, we found that the strongest predictor of participants' prosodic short‐term memory was their earlier prosodic awareness. The direct stress perception measure (DIFFiculty‐diffICulty) accounted for a unique 9% of variance in memory after controlling for age, IQ, and earlier memory skills, the latter of which together accounted for 55% of the variance in the task. This implies that the prosodic patterns of individual words are stored in the mental lexicon, and that participants with more accurate rhythmic stress templates thereby gain a recall advantage, whether they have dyslexia or not. As noted, earlier vocabulary knowledge, a direct measure of lexical development which was matched at the group level, was also a significant longitudinal predictor of prosodic memory. Receptive vocabulary accounted for 7% of unique variance when entered *after* age, IQ, earlier memory skills, and earlier phonological awareness. Taken together, these findings are consistent with the theoretical view that dyslexic teenagers are less proficient at ‘online’ phonological retention of tri‐syllable words compared to CA controls because of impairments in the specification of word forms in the mental lexicon. The most parsimonious interpretation of our data is that the prosodic encoding of lexical forms in long‐term memory is atypical in developmental dyslexia, and that this in turn impairs redintegration processes, leading to poorer short‐term recall of multi‐syllable as well as single syllable words by the same participants (Goswami *et al*., 2013).

## Appendix A Stimulus List for Prosody STM Task

**Table:**

**Same Primary Syllable Stress**			
First Syllable			
succulent	merchandise		vertigo
scandalous	camouflage		utterance
qualify	paranoid		mischievous
optimum	faraway		counsellor
invalid	demonstrate		waterproof
melody	treasurer		clinical
fabulous	prototype		immigrant
masterpiece	colourful		juvenile
resident	notable		masculine
Third Syllable			
disbelief	reassure		comprehend
unforeseen	disregard		entertain
reproduce	immature		overweight
unprepared	indiscreet		rationale
humankind	nationwide		disarray
recommend	silhouette		disappear
whereabouts	millionaire		discontent
underneath	volunteer		represent
indirect	overcome		nonetheless
**Different Primary Syllable Stress**			
			(sequence of primary stress)
bearable	assertive	undermine	1,2,3
infrequent	disagree	extrovert	2,3,1
overdue	celebrate	horrific	3,1,2
lunatic	picturesque	offender	1,3,2
repulsive	aftermath	underfoot	2,1,3
introduce	upheaval	luminous	3,2,1
feasible	respectful	pioneer	1,2,3
enclosure	guarantee	manuscript	2,3,1
incomplete	miniature	pathetic	3,1,2
hesitate	infrared	disloyal	1,3,2
fanatic	soluble	overgrown	2,1,3
indistinct	unfriendly	occupy	3,2,1
complement	barbaric	souvenir	1,2,3
excursion	refugee	lovable	2,3,1
referee	undergrowth	quotation	3,1,2
organise	insecure	misfortune	1,3,2
immortal	arrogant	employee	2,1,3
overnight	dimension	recipe	3,2,1
